# A Multiple Emergency Ventilator as backup solution for disaster situations: prototype development and functional assessment

**DOI:** 10.1007/s11517-025-03395-x

**Published:** 2025-06-16

**Authors:** Aldo J. Suria, Luca G. Paroni, Silvano Seva, Roberto Viganò, Francesco Casella, Alberto Zanella, Giuseppe Baselli, Gianfranco B. Fiore

**Affiliations:** 1 Department of Electronics, Informatics and Bioengineering, Politecnico di Milano, Milan, Italy; 2 Department of Mechanical Engineering, Politecnico di Milano, Milan, Italy; 3 Department of Anaesthesia and Resuscitation, IRCCS Ca’ Granda Ospedale Maggiore Policlinico, Milan, Italy; 4 Department of Cardiac and Thoracic Aortic Surgery, Medical University of Vienna, Vienna, Austria

**Keywords:** Mechanical ventilation, Emergencies, Intensive care units, Acute respiratory distress syndrome

## Abstract

Shared ventilation is an interesting topic amongst researchers due to the possibility of healthcare saturation under disaster conditions provoked by viruses, war environments, diffusive respiratory system diseases, etc. However, general outcomes from this practice have shown several risks. The *Multiple Emergency Ventilator* overcomes these hazards by providing a temporary, pressure-controlled ventilatory support for up to ten patients simultaneously, whilst allowing for a modular and fast assembly during emergencies. This article presents the construction and the assessment of the prototype using patient simulators with different types of bio-mechanical characteristic configurations at ten hospitalization stations. Ventilation parameters at the most distal-to-source station were recorded. Under normal operation, a minute ventilation of 9.6 breaths × L × min^−1^ was obtained. Critical operation during different types of localized sudden pneumatic disconnections was also investigated. Amongst these, only the event of a disconnection of any simulator at its pneumatic connection to the main distribution pipeline highlighted a poor minute ventilation of 4 breaths × L × min^−1^. Yet, the probability of such an event is minimal due to the use of safe mechanical constraints at the relevant pneumatic connections. In this first prototype assessment, the device has shown its capabilities to ventilate multiple patients with consistent pressure behaviours even whilst adverse conditions are imposed.

**Graphical Abstract:**

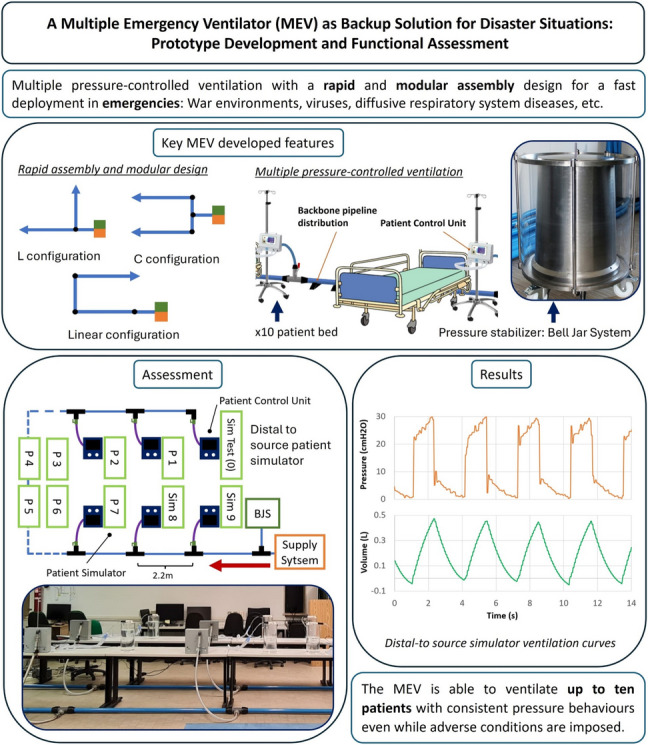

**Supplementary Information:**

The online version contains supplementary material available at 10.1007/s11517-025-03395-x.

## Introduction

The possibility of scarce medical resource conditions has been, for a long time, a topic of interest for the scientific community. Throughout history, disaster situations provoked by rapid infectious diseases [1–3], war environments [4, 5], low-income and middle-income countries scarcity [6], etc. have affected health care systems and tested their preparation and management capabilities. There are multiple variables to control in order to obtain effective management and to prevent such disasters. This paper focuses on the lack of resources at intensive care units (ICU), specifically addressing the crisis raised due to the lack of mechanical ventilators. Different strategies have been proposed to manage the lack of such devices; for instance, sharing ventilation, stockpiling ventilators, and, as a final resource, triage protocols for highest survival rate outcomes [7, 8].

Particularly, during the 2020 SARS COVID-19 pandemic, the ventilation sharing, defined as the ventilation of two or more patients assisted by a conventional unit, was one of the hot topics because of its controversial results [9]. This approach was previously studied by multiple authors [10, 11] to improve the preparation in case of disaster events such as the 1918 influenza pandemic, the 2001 anthrax attacks, and the 2003 SARS outbreak and was extensively investigated during the scarcity of ventilators throughout the 2020’s pandemics [12–16].

Medical societies and regulatory agencies from the USA [17] and EU [18] provided some guidelines to encourage increased production of ventilators and ventilator sharing. However, for this latter technique, those same announcements, specifically the Appendix B from the US entity [17], spotlighted the related hazards, which were investigated in previous articles [19] and reiterated by other statements, pointing out the risks of cross-infection, ventilation impairment, impossibility to manage the Positive End Expiratory Pressure (PEEP) and other issues [20].

The Multiple Emergency Ventilator (MEV) has been designed to overcome these problems and provide an emergency solution, able to provide basic respiratory support for up to ten patients [21]. The main features of the MEV are its rapid deployment, modularity, and intrinsic overpressure security mechanism ensured by its core: the Bell-Jar System (BJS).

The design of the MEV takes a step backward from conventional ventilators, providing a simple, but efficient constant pressure ventilation, which, in case of multiple subjects ventilated in parallel, allows to have an equalized and secure pressure, overcoming the issue of ventilation impairment experienced during previous shared ventilation attempts. The device is intended to be used in emergency situations such as pandemics or endemic disasters, low or low-middle income countries with lack of resources, or any disaster causing the saturation of health care systems. Under these circumstances, the MEV provides a simple, rapid-use and transportable device able to temporally cope with crisis situations. The idea was developed and validated in a computational fashion by Baselli et al. in 2022 [22]. The numerical findings stated that peak inspiratory pressures (PIP) at the most distal patient (25 m away from the source) are diminished by a 6.8% in worst-case conditions, that is, ten patients with synchronized breathing and exerting PIP's in phase, simulating the highest-pressure loss possible. This and further important results from that study showed the feasibility of the MEV concept from a computational point of view.

**Fig. 1:**
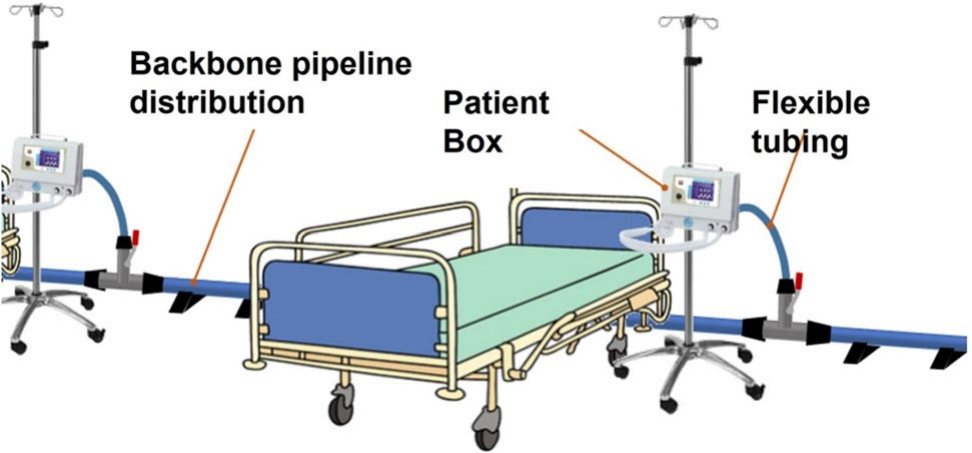
Sketch of a hospitalization station with the MEV installed. The backbone pipeline has rapid plug-in straight or elbow connectors for longitudinal deployment and T connectors for the lateral supply of the gas mixture to each patient. The control unit for each patient connects to the relevant backbone’s T with a flexible tube through a quick safe coupler and a manual safety valve

**Fig. 2:**
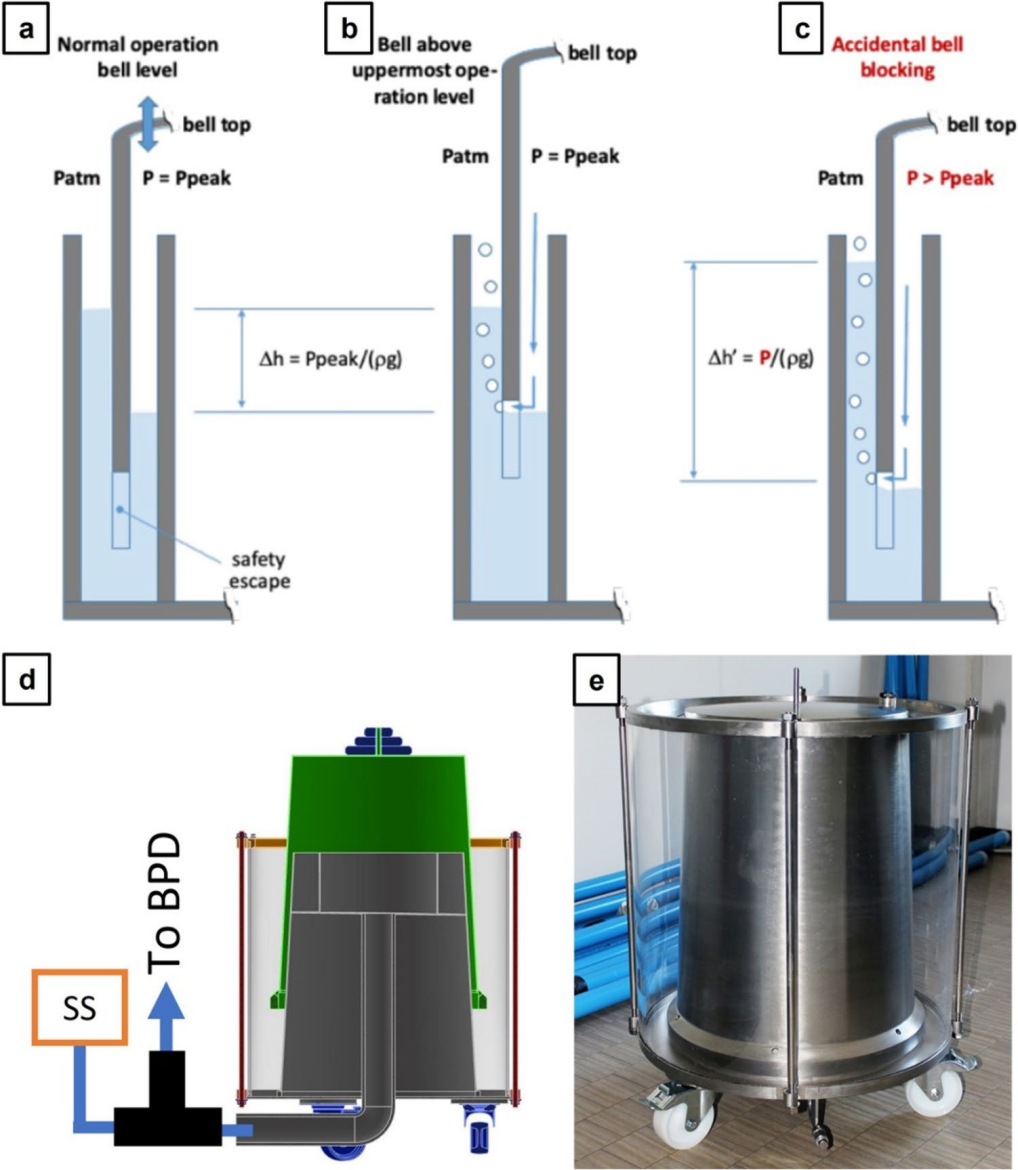
Bell-Jar System (BJS) conceptual idea. **a** The normal operation consents the storage of gas mixture volume at the desired pressure. **b** Gas mixture can be released from the safety escape when the bell reaches its maximum height. **c** Gas mixture can also be released in case of bell blockage by diminishing the inner bell meniscus. **d** CAD section model of the BJS showing its interface with the supply system (SS) and the backbone pipeline distribution (BPD). **e** Final BJS prototype

This article presents the development of a functional prototype of the MEV, detailing design choices and construction features. The device was then subject to experimental tests and validation focusing on the maintenance of physiological pressures and tidal volumes (TV) using an in-vitro bench test. Elements for the construction were chosen with the scope of enabling easy assembly and disassembly, thus yielding its modularity and transportability. An overview of the macro-components is described hereafter:

- *Medical gas supply system (SS)*: throughout this work, it will be assumed that the premises where the MEV is deployed have a medical gas plant available, capable of ensuring the necessary supply of ventilation gas mixture for ten patients.
- *Pressure stabilizer*: also called BJS, it is the component responsible for maintaining a constant pressure at the backbone distribution. The design of this element is focused on the intrinsic safety of the patients allowing multiple security mechanisms (visible in Figure 2).
- *Backbone pipeline distribution (BPD)*: low pressure loss pipeline distribution supplying the gas mixture to each patient is visible in Fig. 1.
- *Patient control units (PCU)*: at each hospitalization station, a control unit allows the user to manage the single patient ventilation and to obtain data functional to monitor patients’ breathing parameters (visible in Fig. 1).

## Materials and methods

The present section focuses on the construction of the prototype itself and on the design of a test bench to assess the functionality of the device.

### MEV prototype construction

To build a prototype and test its functionality, many aspects of the machine were simplified. The most important assumptions are the following:

- Ambient air as fluid under test
- At low pressures air can be treated as an incompressible fluid
- Usage of non-medical grade materials was permitted for the construction of macro-components, provided that the medical-grade counterparts were available on the market
- Patients are assumed to be fully curarized; i.e., each patient’s respiratory system biomechanics can be simulated as a passive RC circuit

Each macro-component was first constructed and tested separately. This subsection breaks the construction of the system as it was mentioned in the introduction.

#### Bell-Jar System

The Bell-Jar System (BJS) is the core of the system as it allows to stabilize and set a constant pressure depending on the bell’s weight plus additional weights used to increase the pressure magnitude. The BJS was designed to work in a nominal pressure of 30–31 cmH2O. During gas mixture supply, the bell top moves in a vertical manner, storing volume at a (theoretical) constant pressure as shown in Fig. 2a. A position sensor is used to detect the vertical movements of the bell and is used as a feedback signal to control the gas mixture source inflow and thus maintaining a stable bell height. However, the main feature of this element is its intrinsic overpressure security mechanism given by the safety escape shown in Fig. 2a. When the bell reaches its height limit, gas can escape through this aperture as shown in Fig. 2b. Additionally, the system is also protected in case of an accidental bell blocking. The overpressure generated depends on the height at which the bell top blocks, and it never exceeds 43 cmH2O. In this case, the pressure generated displaces the meniscus between the bell and jar, allowing for gas to be released through the safety escape as shown in Fig. 2c.

**Fig. 3:**
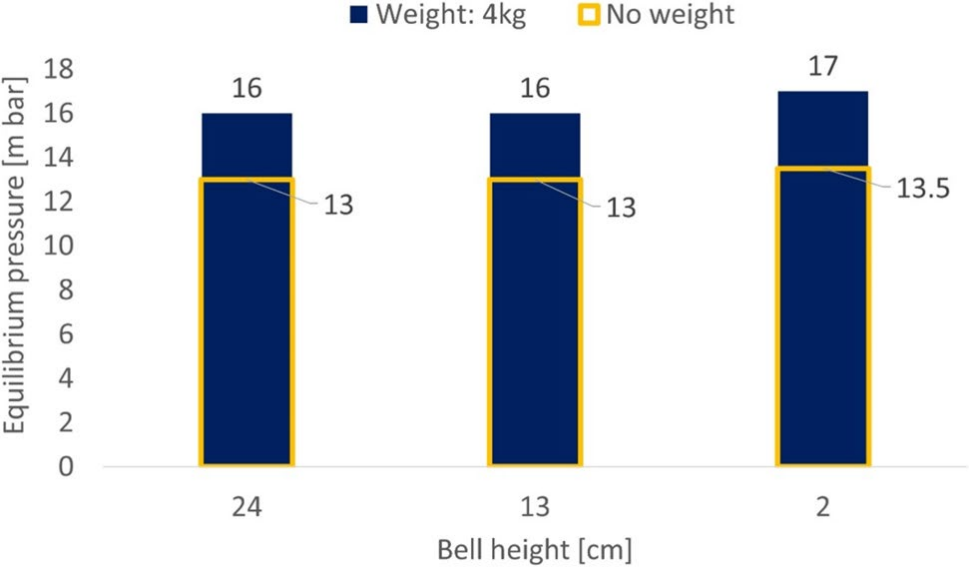
BJS testing of pressure maintenance at different Bell-height levels with no additional weight applied or with a 4-kg additional weight applied

To test the functionality of the BJS and assess whether the device was able to sustain the configured pressure, a test for different bell-height was realized. The pressure inside the BJS for different heights and with and without weight is reported in Fig. 3.

#### Backbone pipeline distribution

A key characteristic of the MEV is its modularity, rapidity and versatility to adapt to the physical space where it is mounted. This is addressed by the rapid mount and dismount feature of the backbone pipeline distribution (BPD). The latter transports the mixture from the SS/BJS complex towards each hospitalization station. To increase the flexibility of the system, each PCU is connected to the BPD with a flexible tube.

**Fig. 4:**
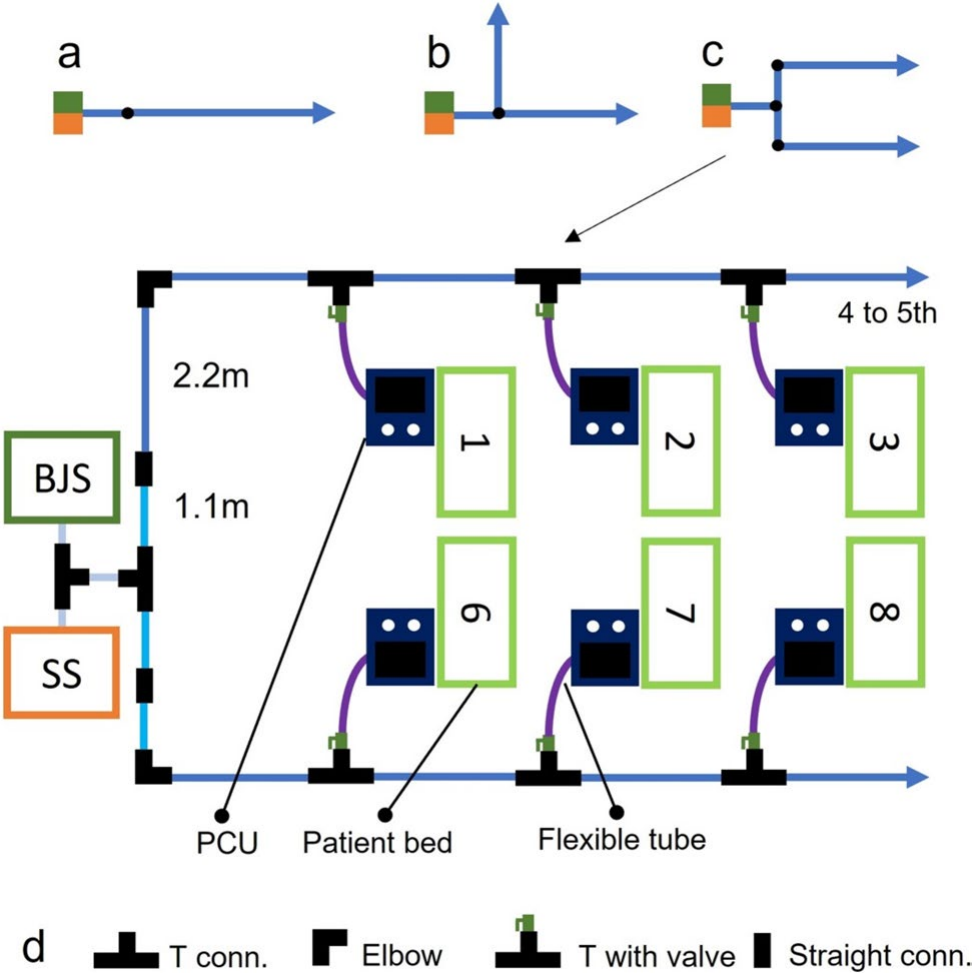
Layout examples of the BPD. **a** Linear configuration. **b** “L” configuration. **c** “C” configuration. Blue, cyan and grey solid lines indicate 2.2-m, 1.1-m and 0.6-m rigid tube modules. **d** Scheme legend

The BPD was constructed with a rapid assembly pipeline system (AIRNET® System Aluminium, Internal diameter: 50 mm, MultiAIR Italia S.r.l—International, Torino, Italy). The system allows to connect different rigid tube modules by means of rapid plug-in airtight connectors. Three types of tube modules were created with the following lengths: 2.2, 1.1 and 0.6 m. The module with the major length maintains enough patient’s bed distance, whereas the other two module sizes allow a modular construction when curves or different layouts are needed to adapt to the room dimension. To exemplify the latter statement, a series of layouts are proposed in Fig. 4.

At each hospitalization station, the BPD has a T connector, whose lateral branch features a manual tap with a safety mechanism which avoids unintentional manoeuvres. Each derivation supplies the gas mixture to one individual patient. Each PCU is connected to the closest BPD derivation with a flexible tube (DEMER, Italy), whose orientation further increases the flexibility of the system layout. The connection is made with a quick coupler endowed with an anti-disconnection leverage. These tubes are made of braided stainless steel and have an ID of 3/4 of an inch with a length of 1.5 m.

#### Patient control unit

**Fig. 5:**
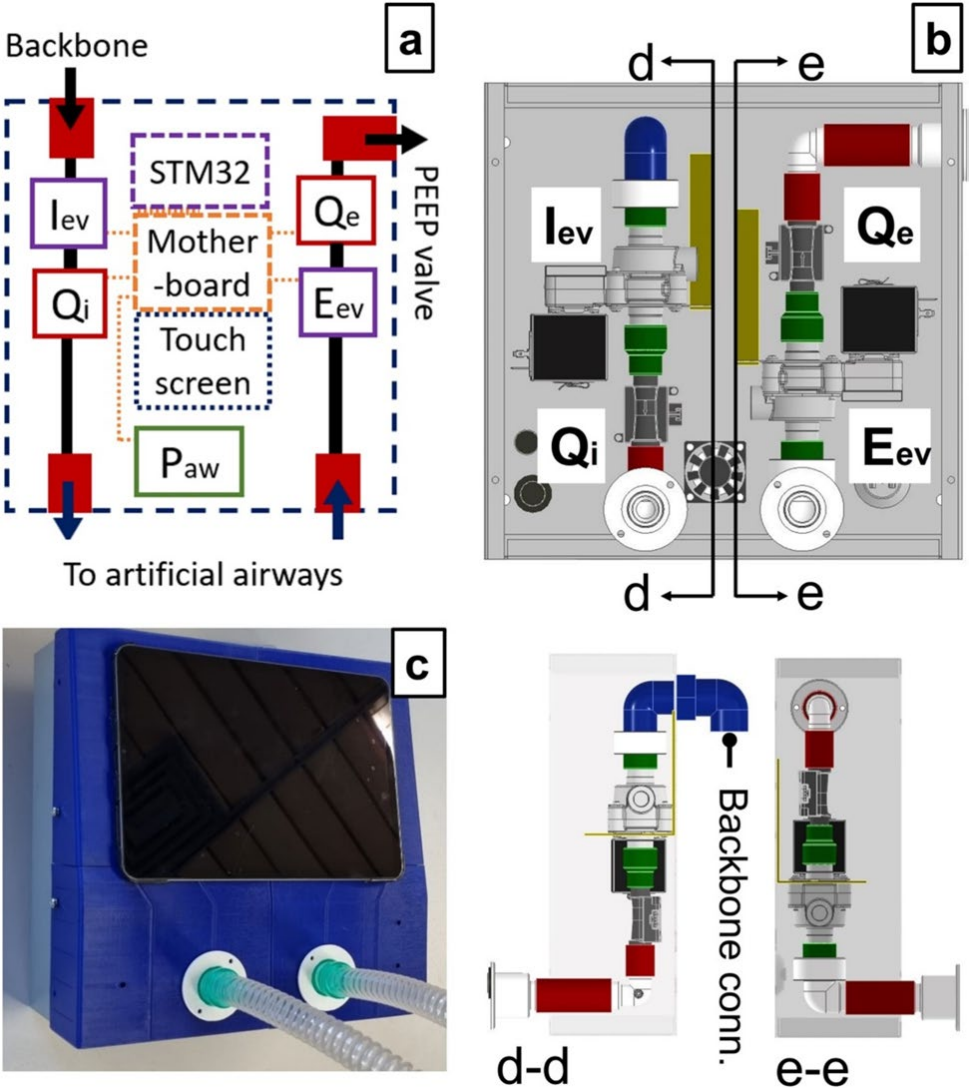
**a** Patient control unit (PCU) schematic; black solid lines represent internal pneumatic lines, whereas orange dotted lines show electric connections. **b** PCU open case showing each element and respective cross-section views. **c** Final PCU realization

Ten PCUs were constructed using the general scheme from Fig. 5a, and a detailed pneumatic circuit is presented in Fig. 5b. The chassis is made of mild steel, which protects all elements inside the case, whereas the frontal cover is 3D printed. Each PCU contains the following elements:

- Inspiratory and expiratory solenoid valves—ASCO series D132 (Emerson Electric Co., St. Louis, Missouri, USA): The inspiratory (Iev) and expiratory (Eev) solenoid valves allow the closure and opening of the inspiratory and expiratory branches respectively. Such normally closed valves are actuated with a 24 VDC signal.
- Flow sensors—FS1015 CL (Siargo Ltd, Santa Clara, California, USA): Placed downstream of the Iev and Eev, they allow to measure the inspiratory and expiratory flow (Qi and Qe respectively) and therefore provide an indirect measurement of the TV.
- Pressure sensor—MPX5010DP (NXP Semiconductors N.V., Eindhoven, Netherlands): Placed downstream of the Qi, it allows to measure the airway pressure (Paw).
- Micro-controller STM32 F429I-DISC1 (STMicroelectronics NV, Plans-les-Ouates. Geneva Switzerland): it processes the sensors’ signals and provides the control for the opening and closure of the solenoid valves. This element also allows the control of a user interface for the PCU.
- Tactile display—NX1060P101-011 C-I (Nextion, Shenzen, Guangdong, China): Touchscreen displays the user interface and sending commands to the micro-controller.
- Patient disposable standard equipment: This includes a PEEP valve, breathing circuit, humidifiers and an Endo-tracheal tube (ETT).

For the sake of simplicity, a simple time-cycled control of the opening and closing of solenoid valves is implemented using the micro-controller. Table 1 presents a cost estimation of each macro-component mentioned in this section.

**Table 1:** Prototype cost estimation of the MEV for a tenfold patient configuration

Prototype part	Cost (EUR)
Backbone pipeline distribution	2300
Bell-Jar System	4200
10 × patient control units	3300
10 × Disposable standard equipment	300
**MEV total cost**	**10100**

### MEV prototype resting

A test bench was constructed to assess the system functionality. To this end, the SS was substituted for an air-supply system simulator (AUXSS) able to furnish 10 patients with the respective in-rest volume intake. Details are reported in the supplementary materials.

Likewise, to mock the presence of patients, functional passive lung simulators were designed to replicate different pathological bio-mechanical features found in acute respiratory distress syndrome (ARDS) patients. A simple RC circuit representing the airway resistance (Raw) and the respiratory compliance (C) was used to model the simulators. To simplify the variety of conditions presented in ARDS patients and to test the MEV with different respiratory system characteristics, three typical values of C and Raw were chosen, ranging from mild (1), severe (2) and critical conditions (3), as shown in Table 2.

**Table 2:** Biomechanical parameter distribution for ten patient simulators

Resistance [cmH2O*min/mL]		Compliance [mL/cmH2O]		
		C1	C2	C3
		20	40	60
R1	**8**	P1	P2	P3, P0
R2	**12**	P4	P5	P6
R3	**15**	P7	P8	P9

To fulfil all values and allow an easy combination between elements, nine possible combinations of C (C1, C2, and C3) and raw (R1, R2, R3) were built to get a set of passive patient simulators representing the discrete values shown in Table 2. More details on the construction and working principle of the RC simulators can be found in the supplementary materials.

## Results

**Fig. 6:**
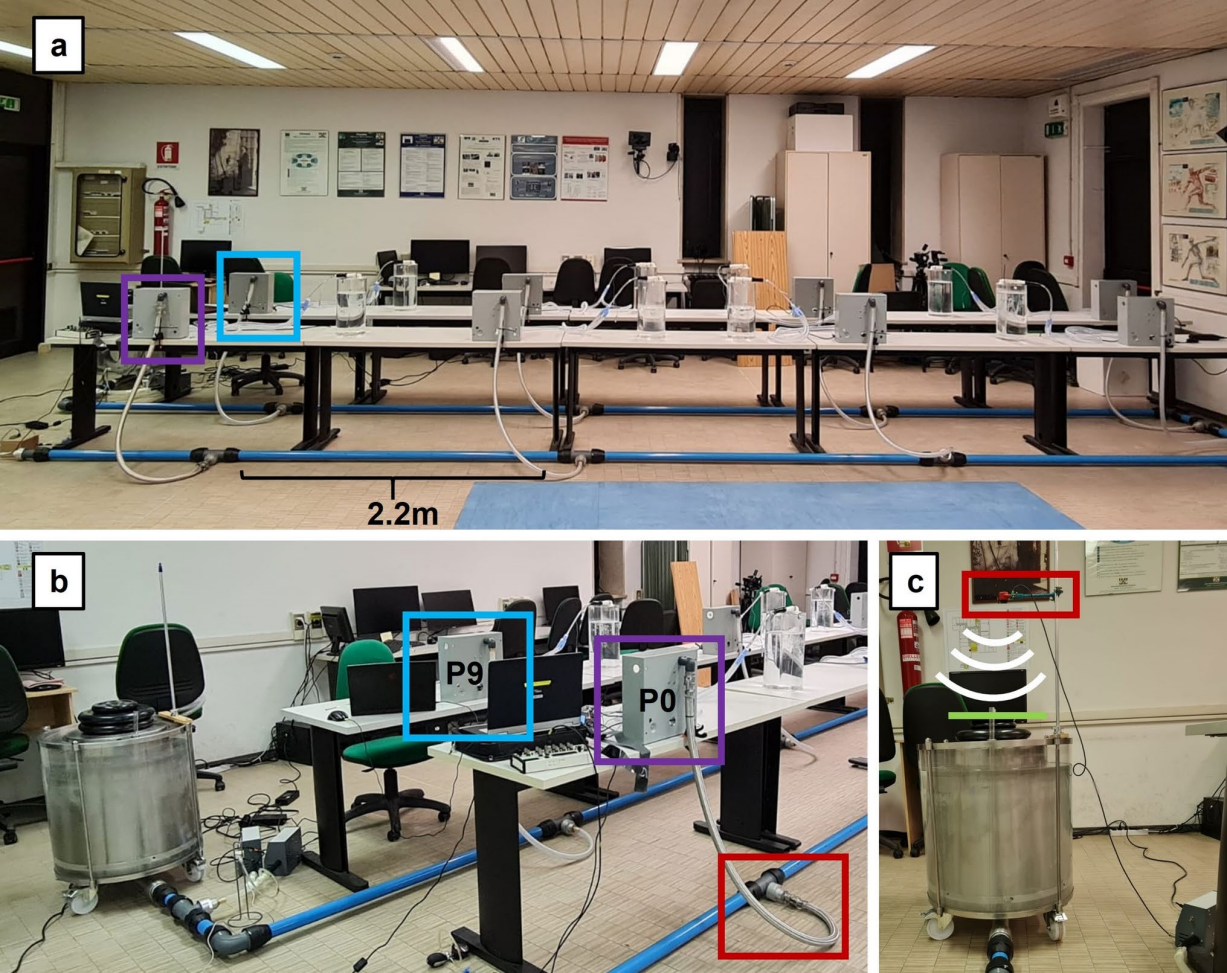
**a** MEV functional prototype tested via patient simulators. Each PCU is separated by 2.2-m modules. A linear “U” layout was tested to check the ventilation quality at the most distal patient (P0—purple rectangle). **b** BJS and initial part of the BPD, the most proximal patient (P9—blue rectangle). **c** Ultrasound detector of BJS bell height

**Fig. 7:**
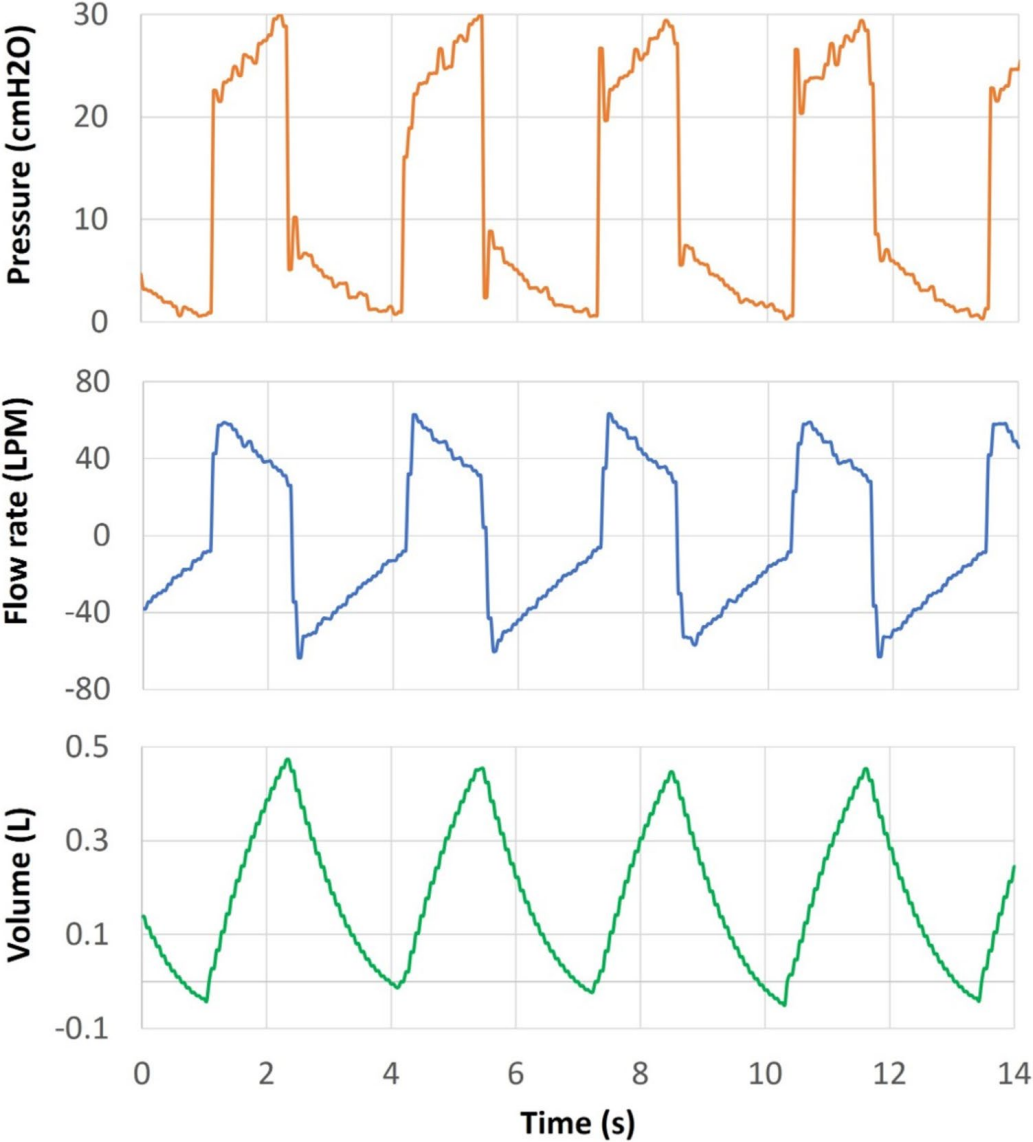
Curves at P0 under the normal operation of the MEV. P0 achieved an average TV of 0.48 L, resulting in a minute ventilation (MV) of 9.6 breaths × L × min−1

The final assembly of the whole system to be tested is presented in Fig. 6, and Fig. 7 shows the flow rate, pressure and volume curves at the most distal patient (P0), which in the linear configuration is placed downstream of the BPD, representing the worst-case scenario in terms of pressure loss due to pipeline length.

The normal operation test consisted of recording the ventilation performance at P0 under the following conditions:

- Ambient air as fluid under test
- BJS set to 30 cmH20 (22 k g added to the top of the Bell-Jar)
- Asynchronous patients breathing cycles
- Patient’s simulator bio-mechanical characteristics were distributed according to Table 1.
- Time cycled control with the parameters:
  - Inspiration to expiration ratio (*I*/*E*) set to 1/1.5
  - Breathing frequency 20 breaths × min−1

Under these conditions, the airway pressure was maintained to 30 cmH2O thanks to the BJS action, as shown in the pressure curve in Fig. 7. Moreover, the critical patient achieved an average TV of 0.48 L, resulting in a Minute Ventilation (MV) of 9.6 breaths × L × min−1.

To push even further the system, the same assessment was performed in the following critical conditions.

- BJS critical condition (Appendix Figures 8, 9, and 10 respectively)
  - Over pressure safety mechanism action
  - Rapid filling of the BJS
  - Emptying of the BJS
- Sudden tube disconnection scenarios (Appendix Figures 11, 12, and 13 respectively)
  - Endotracheal tube disconnection
  - Breathing circuit disconnection
  - Flexible tube disconnection
- Patient synchronization scenario (Appendix Figures 14 (full ventilation curve time frame) and 15 (zoom in into two-time frames))
  - All patients performing synchronized PIP

See the Appendix for the ventilation outcomes at P0 under these conditions.

**Fig. 8:**
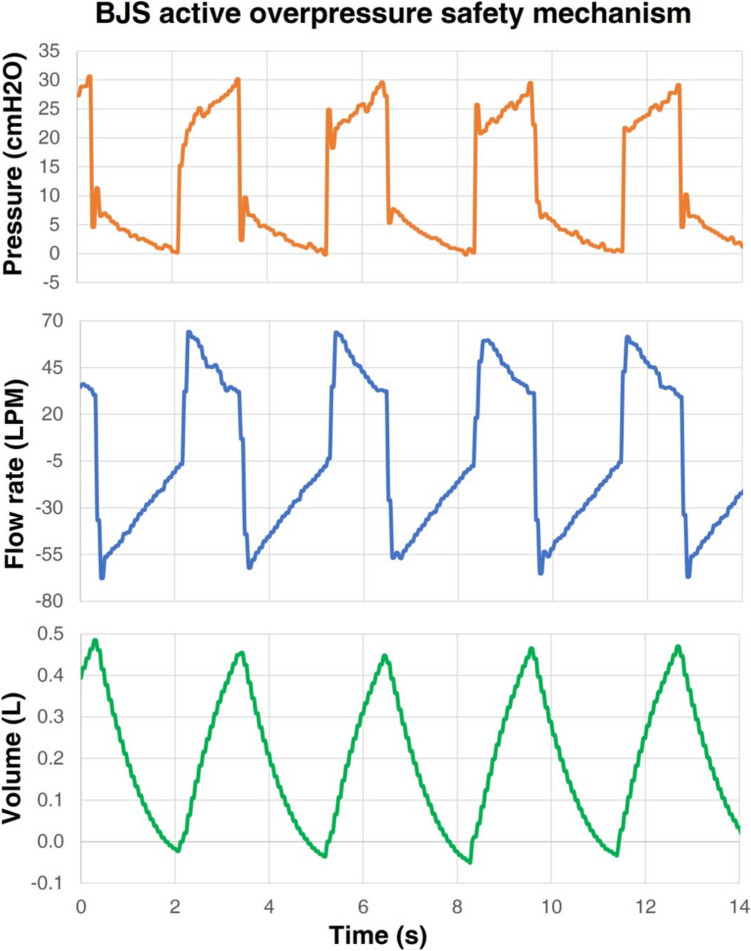
P0 ventilation curves during the overpressure mechanism action

**Fig. 9:**
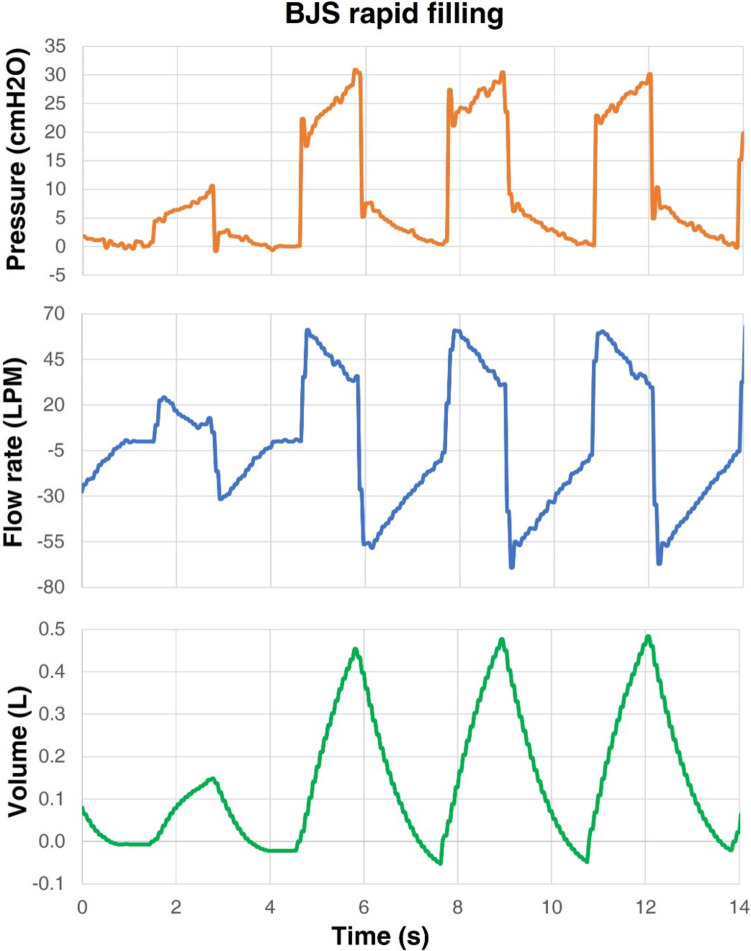
P0 ventilation curves during the filling of the BJS. The initial pressure increases from 0 to 30 cmH2O as the bell height rises

**Fig. 10:**
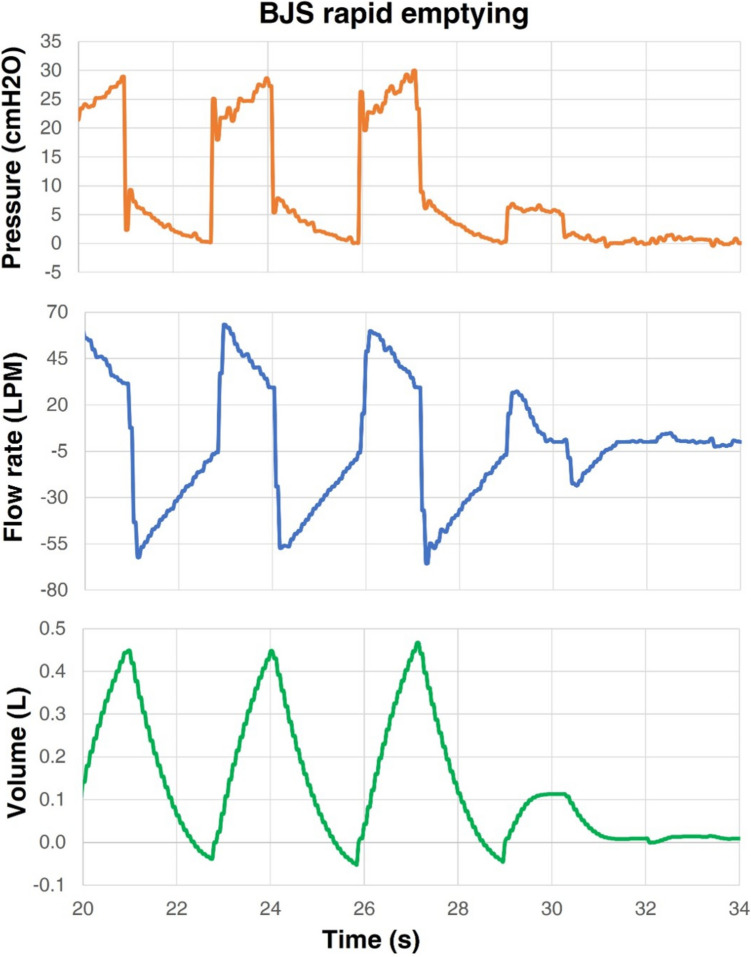
P0 ventilation curves during the emptying of the BJS. The pressure in the BJS decreases until reaching 0 cmH2O as the bell height lowers

No over or under pressure was recorded when the BJS achieved its maximum volume or bell height (Fig. 8), yet strong air escape took place in the form of bubbles causing water inside the BJS to spill in small quantities. On the other hand, when the BJS was tested during the filling phase, an increase of the PIP up to 34 cmH2O was reached (Fig. 9), although during further cycles the PIP behaviour stabilized again back to 30 cmH2O. During the emptying of the BJS, a decrease in PIP took place to about 28 cmH2O in the first 3 breathing cycles; after this, the ventilation decreased considerably until losing all positive pressure (Fig. 10). This scenario is equivalent to the gas mix source being cut off.

**Fig. 11:**
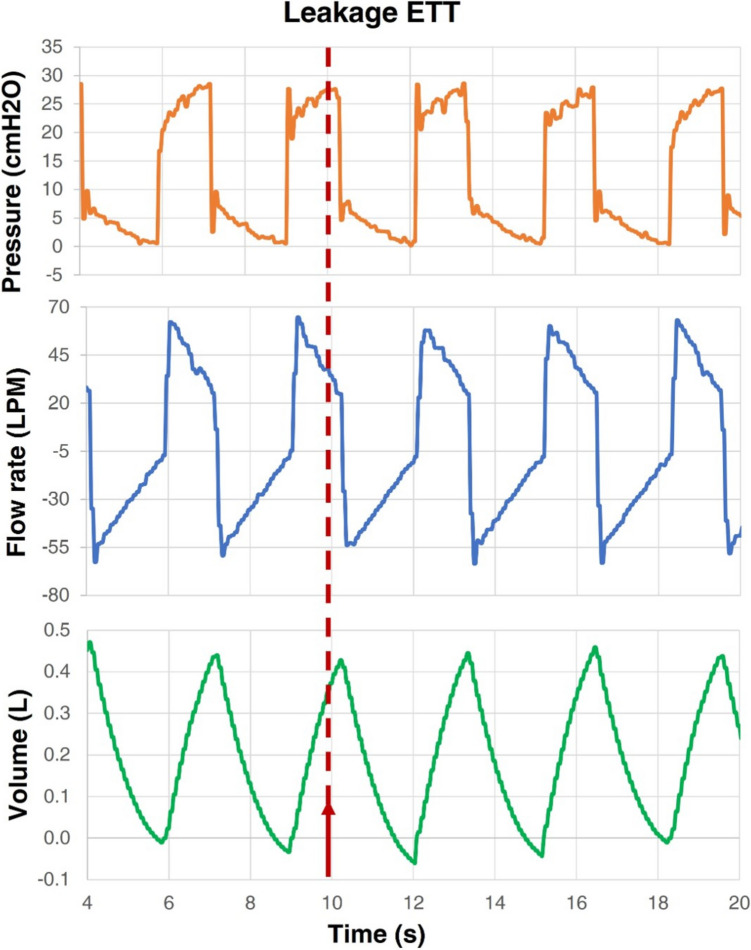
P0 ventilation during sudden disconnection of the ETT localized at the patient 1 (proximal to P0). The event is performed at time = 10 s

**Fig. 12:**
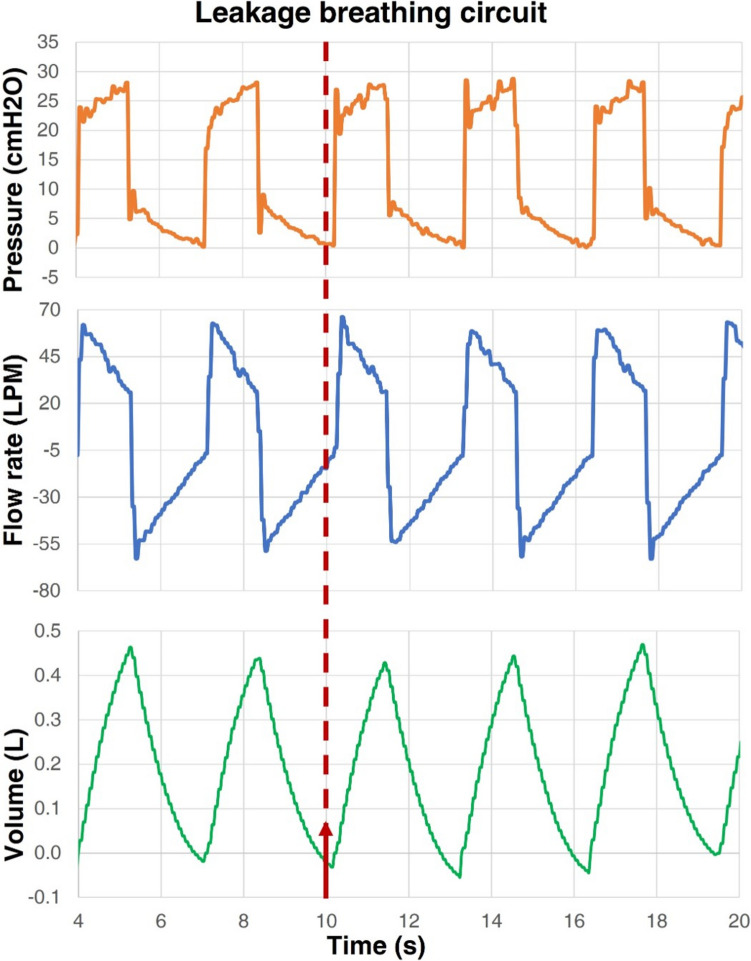
P0 ventilation during sudden disconnection of the breathing circuit localized at the patient 1 (proximal to P0). The event is performed at time = 10 s

**Fig. 13:**
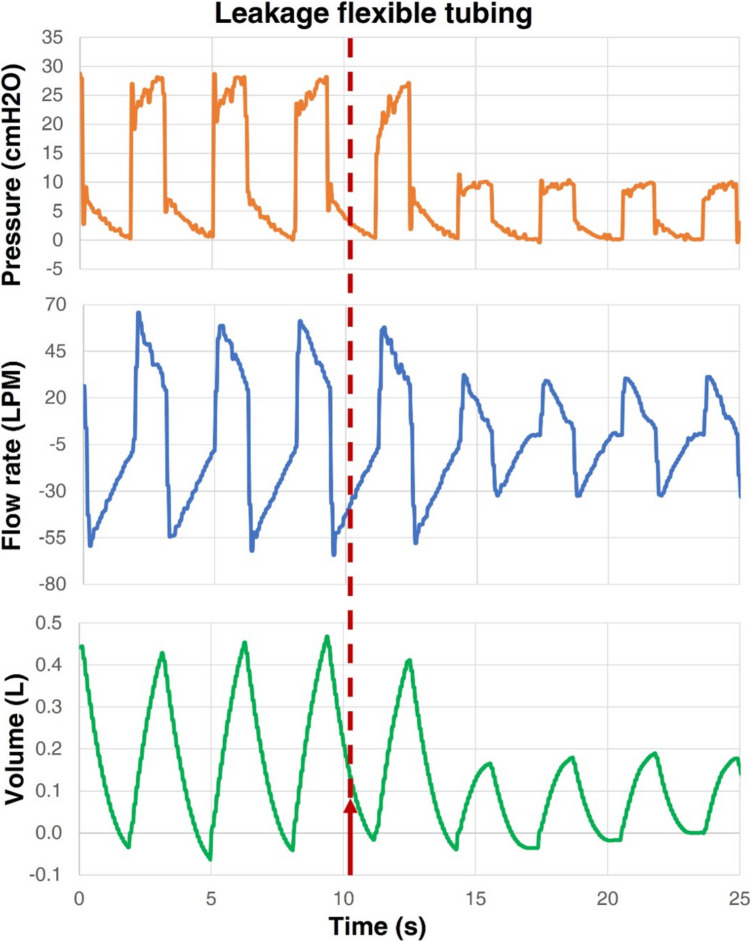
P0 ventilation during sudden disconnection of the flexible tube localized at the patient 1 (proximal to P0). The event is performed at time = 10 s

Leakage tests were performed from P1 to P9. There were no appreciable changes between one or the other; thus, only results from localized events at the most proximal to P0 (P1) are considered. Disconnections from the ETT or breathing circuit did not sensibly affect the ventilation of the patient under study, as shown in Figures 11 and 12. However, a strong mismatch of ventilation was appreciated in the case of disconnection of the flexible tube from the dorsal (Figure 13) with PIP at P0 falling to 10 cmH2O and the minute volume reduced to 4 breaths × L × min−1.

**Fig. 15:**
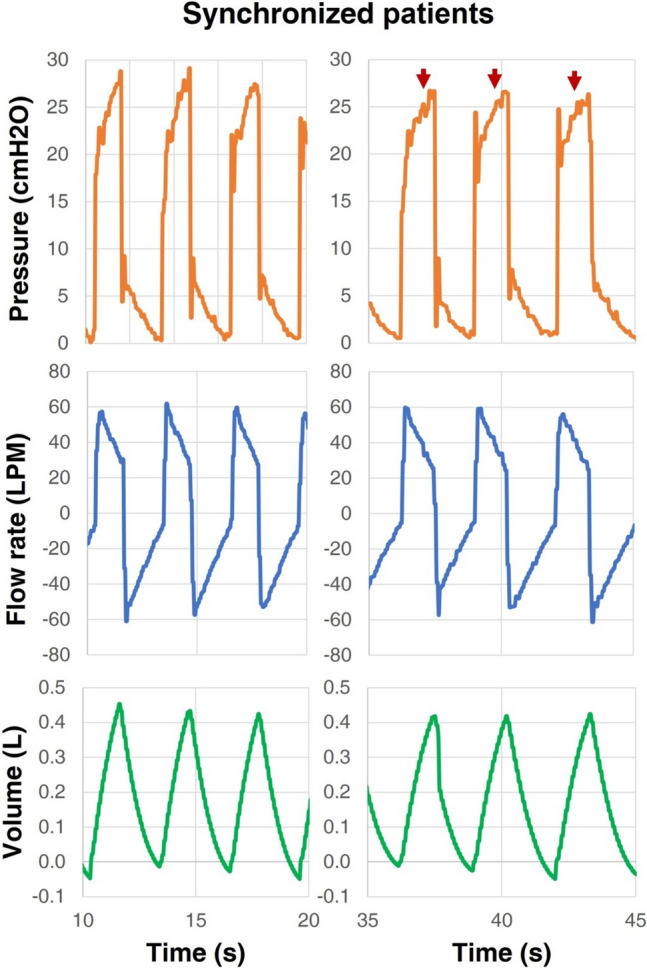
Zoom-in into two-time intervals during synchronized breath condition. From 10 to 20 s and from 35 to 45 s. At these time intervals, a decrease in pressure can be appreciated, suggesting an increased pressure loss due to breathing cycles in phase

Lastly, to test the MEV under the condition of perfectly synchronized breathes, the PCUs were activated sequentially, separated by intervals of 10 s (Figure 13). This condition caused, in some cycles, a decrease of pressure to 27 cmH2O, as shown in Figure 15.

## Discussion

A functional prototype of a Multiple Emergency Ventilator (MEV) has been built and evaluated in terms of pressure and TV starting from the initial conceptual idea and computational study by Baselli et al. [22] stressing areas of improvement using commercially available products. The development of this prototype is a proof that, by adopting a basic constant pressure ventilation, temporary emergency support to several subjects in parallel is feasible not only in theory but also in practice. Although many literature articles associated multi-subject ventilation with ventilation sharing, the MEV does not depart from the modification of a conventional design planned for single patient ventilation, but from an original, systematic analysis of the design requirements for a system capable of ventilating multiple patients. These requirements emerged recently during the COVID-19 pandemic, and thus, ventilation sharing was re-proposed as a viable emergency solution [17]. Observations such as ventilation impairment due to different respiratory mechanics [12] or high increase of volume and pressure due to abrupt ventilation circuit changes (e.g. endotracheal tube occlusion) [13] pointed out the fragility of this technique. Even though further works successfully achieved shared ventilation proof of concept, either by simulation [14, 15] or direct human experience [16], the procedure was still regarded as complicated, risky, and falling out of the intended use of single ventilators [9]. Many low-cost easy-to-build non-invasive ventilators (NIV), comprising commercially available components, were proposed during the pandemic crisis with prices lower than 75 USD [23]. However, in case of sudden intubation [24], such devices might not be sufficient to provide a proper ventilation. The MEV aims to tackle these issues with a novel design approach, by (i) uncoupling the fluid dynamics between patients, (ii) specifically and correctly designing pressure and flow for the accounted number of patients, (iii) avoiding cross-contamination, and (iv) incorporating a PCU for each bed, allowing to set single-patient parameters. Costs of building this prototype have been estimated to be around 10 k. Such a level of expenditure might notably reduce switching from prototype realization to a serial production, reaching reasonable levels for a system which might become a standard, safe and reusable piece of equipment carried by first-aid emergency teams towards disaster areas. Indeed, with respect to other low-cost solutions, a fundamental strength of this system is its intrinsic security against barotrauma for invasive mechanical ventilation, thanks to the integration of the BJS pressure stabilizer/limiter. This macro-component has shown its capabilities to keep safe conditions throughout all operations and to be easily adjustable by simply adding weights at the top of the bell. This might be particularly crucial in scenarios wherein the aim is to at least increase the overall probability for patients to survive. These scenarios include pandemic situations as well as natural disaster or war scenarios. In case of automatic system failures (e.g. electronic failure and Bell-Jar blockage) the system is designed to be intrinsically safe as the unwanted air volume increase would be released due to the meniscus air escape mechanism shown in Fig. 2. During the BJS over-pressure air escape event, no over-pressure or alterations were recorded at P0. It is paramount to highlight the features of the BPD, which allow the mounting of the system in a rapid fashion. Deployment of tubes and assembly of PCU, BJS and other elements took no more than 4 h, considering the participation of 2 workmen. Under emergency conditions, the layout can be adapted to the room dimensions, as shown in Fig. 4, and then deployed to be used in a few hours.

With respect to the preliminary design, which was focused on showing the theoretical feasibility of the multiple ventilation approach, some aspects have been integrated and improved in the real prototype:

- Quick-coupling modular tubing for the BPD: Previously, screw-able mechanisms connecting the modules of the BPD were proposed. In this work, though, the use of quick coupling mechanisms based on press-fit solutions using radial O-ring sealings has been explored. The experience with this type of coupling has shown to ensure an extremely easy and fast mounting procedure.
- Quick-coupling flexible tubing: Connections from the BPD to each PCU were accomplished using flexible stainless-steel tubing, which enhances the organization in space of each patient’s station. The previous proposal was to use vertical straight-up poles that can be connected to each PCU.
- BJS height level sensing mechanism: inlet flow from the BJS is controlled by a position sensor which sends a feedback signal to the flow actuator.

Comparing the measurements obtained in the present work with the previous computational results, Baselli et al. reported PIP decreases of up to − 6.8% during asynchronous normal operation [22]. During the 30-s test of the MEV functional prototype, asynchronous normal operation showed a decrease in PIP of P0 of up to − 7%, showing good agreement between the two studies. Furthermore, values of Fig. 7 reported good TV and MV without over pressure. It must be considered that the MEV topology adopted for this test represents the worst-case scenario in terms of pressure losses due to length. Other parallel layouts which split the BPD into two or more branches will have even lower pressure losses. This guarantees that under normal operations, any layout of the MEV can be used to adapt to the room space limitations.

Sudden leakage test also has showed that leakage events at a certain patient do not sensibly affect the ventilation of other patients in the proximity, as far as an ETT or breathing circuit disconnection are concerned. As mentioned before, these leakages are not rare due to the simple press-fit coupling designed for mating female and male connectors. A leakage at the backbone lateral derivations, instead, was shown to be of high risk to every patient in the system. This event, however, is less probable to happen since the flexible tube, connecting the PCU to the BPD, was endowed with a special quick coupler, which is hard to disconnect by accident, plus a mechanical sphere tap that is intended to intervene on this type of events.

Finally, tests performed in synchronized conditions have shown a decrement in some peaks of the PIP, suggesting that in case of combined peak inspiratory flow rate, the pressure losses due to the BPD length cease to be negligible. This result marks the limit of the MEV, as using a worst-case scenario linear layout combined with breathing cycles in phase could affect the quality of respiratory assistance at the most distal patient. Therefore, it is imperative to avoid such combination.

## Conclusion

The conceptual idea of a Multiple Emergency Ventilator has been implemented; a prototype has been designed, built and tested, focusing on the strength points and the interesting to-verify conditions. The MEV is intended to gain time during emergency situations by maintaining a constant pressure ventilation in up to ten patients when conventional ventilators are no longer available. The system’s design allows it to be rapidly assembled in multiple layout architectures, favouring a rapid impact in case of health care system saturation. Recent years’ experiences have shown the saturation conditions involving the need for ventilating several patients simultaneously may occur either in low-middle income countries or high-income countries.

Our experimental bench test results encourage the potentiality of this emergency system as a possible solution that can be adopted by hospitals, when intensive care units are saturated, as well as by non-profit organizations which are often operating in complex scenarios where the first crucial goal is to at least increase the survival probabilities of patients. The ventilation performance under multiple limit conditions has shown the solid points and risks of the system, accentuating the robustness of the Bell-Jar System and the negligible pressure losses generated in the backbone pipeline distribution. On the other hand, the risk of poor ventilation in all patients due to flexible tube disconnection has been exposed. However, since this issue was expected, the risk was mitigated by providing the system with safe quick coupling connectors and mechanical valves, allowing it to rapidly stop the flow in case of leakage. In case of a perfectly synchronized patient, risks of low PIP values have been registered; therefore, linear MEV layouts should be avoided. Other risks to be mitigated are the laying of the BPD on the ground and the effect of high bell oscillations on normal operation. Future developments involve, firstly, the substitution of the generic elements by their respective medical grade. Testing the system using an oxygen source or oxygen concentrator able to supply the correct amount of flow requested by 10 patients, and finally, the addition of a vent line and its further testing to avoid cross-contamination.

## Supplementary Information

Below is the link to the electronic supplementary material.

Supplementary file1 (PDF 444 KB)

## Figures and Tables

**Fig. 14:**
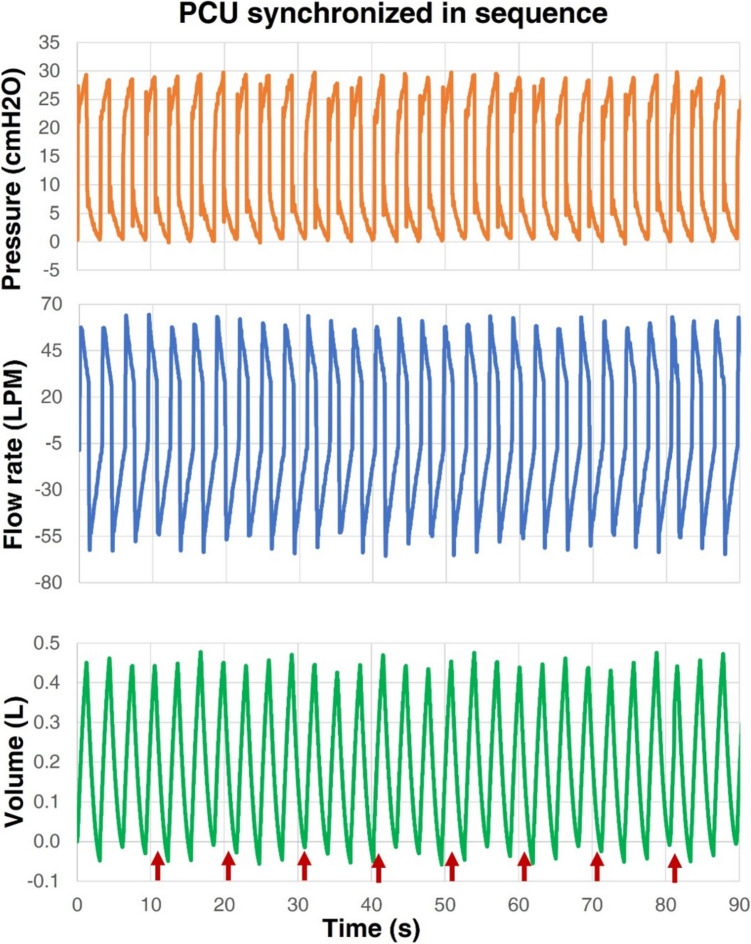
P0 ventilation during the sequential activation of each PCU ventilation control. Every 10 s, the breathing cycle of a new patient simulator is performed

## Data Availability

Data are available upon request.

## Appendix

Ventilation trends obtained at P0 under critical conditions mentioned in the “Results” section are reported (Figs. 8, 9, 10, 11, 12, 13, 14 and 15).

## Code availability

Firmware from control units is available online (See supplementary materials).
