# Supplementary material for: A Multiple Emergency Ventilator as backup solution for disaster situations: prototype development and functional assessment
Source: Med Biol Eng Comput. 2025 Jun 16;63(11):3285–303. doi: 10.1007/s11517-025-03395-x (PMC12634770; doi:10.1007/s11517-025-03395-x)
Supplement: Supplementary file 1 — Supplementary file1 (PDF 444 KB) [file 11517_2025_3395_MOESM1_ESM.pdf]

Article title: A Multiple Emergency Ventilator as Backup Solution for Disaster Situations: Prototype Development and Functional Assessment

Journal name: Medical & Biological Engineering & Computing

Author names: Aldo J. Suria[1,4*], Luca G. Paroni[1], Silvano Seva[1], Roberto Viganò[2], Francesco Casella[1], Alberto Zanella[3], Giuseppe Baselli[1], Gianfranco B. Fiore[1].

Affiliation: [1] Department of Electronics, Informatics and Bioengineering, Politecnico di Milano, Milan, Italy

[2] Department of Mechanical Engineering, Politecnico di Milano, Milan, Italy

[3] Department of Anaesthesia and Resuscitation, IRCCS Ca' Granda Ospedale Maggiore Policlinico, Milan, Italy

[4] Department of Cardiac and Thoracic Aortic Surgery, Medical University of Vienna, Austria

***Corresponding author e-mail:**
aldo.suriaroldan@meduniwien.ac.at

Supplementary materials

Air-Supply System Simulator

An auxiliary air-supply system simulator (AUXSS), able to furnish the whole system was designed using two blower control units as shown in figure 1. Each control unit (Fig. 2) consisted of a centrifugal air blower (DELTA Electronics Inc, Taipei, Taiwan) and were capable to produce a flow of 240 L/min at 30 cmH2O at maximum speed. The feedback signal provided by the BJS position sensor (Baumer Electric AG, Frauenfeld, Switzerland) was used to control the air flow supplied by the AUXSS, allowing to set and maintain the working point of the bell height. A PID controller implemented in an STM32 controller was used to this scope.

The following links contain an open access site to the PCU and BJS feedback controller unit drawings and to the firmware used with the STM32 controller.

- MEV boards: https://github.com/silseva/MEV-board
- MEV firmware: https://github.com/silseva/MEV-firmware

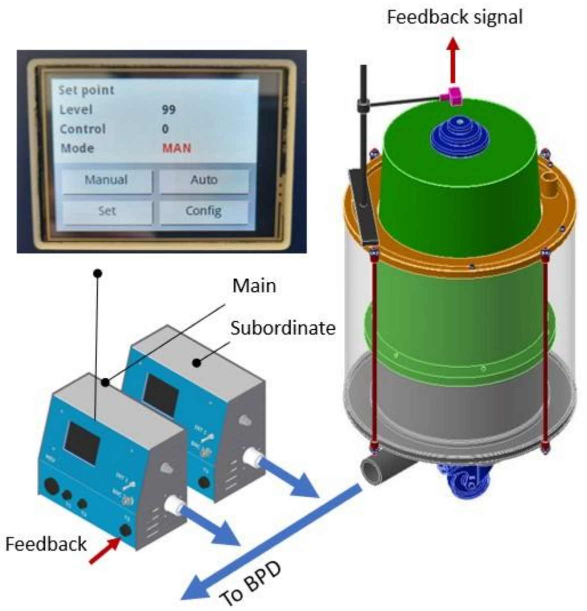

Fig. 1 Double blower source supplying air to the BPD, since the source is connected to the BJS the overall BPD pressure maintains its configured pressure. A feedback signal sent to the Main blower allows to maintain a constant bell level height. In addition, the general menu for the operation is shown in the image, where the user can decide between a manual or automatic operation and can set the desired level height or configure the PID controller used for the automatic control.

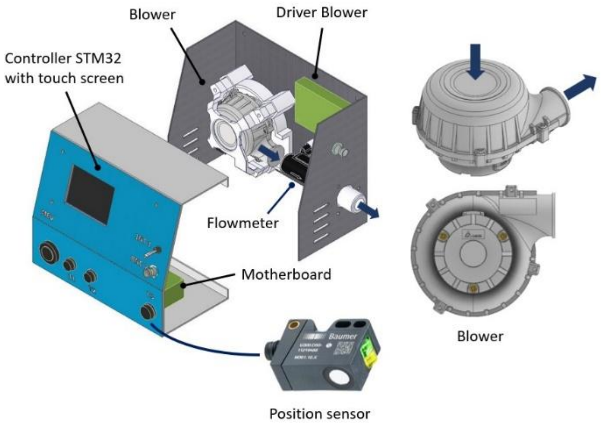

Fig. 2 Scheme of a blower control unit integrating a position sensor. A unit can be used as a subordinate to the Main Blower, which acquires and process the position signal.

Patient simulator design

The combination in series of the Raw and C was used as a patient simulator model. Details on respective design follow. Figure 3a shows the combination and addition of the breathing circuit and an endotracheal tube (ETT). These elements were added to take into account the additional resistance introduced in a typical intubated patient.

Respiratory compliance model

The compliance model working principle was based upon Stevin's law applied to two communicating cylinders as shown in figure 4. In the initial condition figure 4a atmospheric pressure is applied to both water surfaces, the internal (Ai) and the external minus internal cylinder area (Ae – Ai).

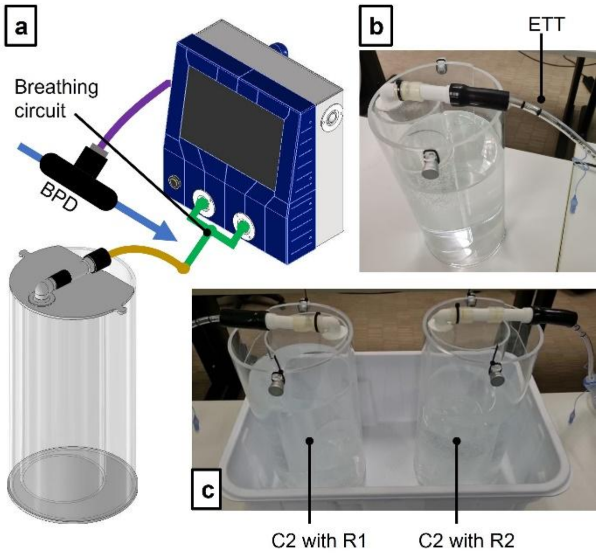

Fig. 3 Example layout of the connection between a PCU and a patient simulator (a). Patient simulator, showing the ETT connection attached to a flexible tube (Black tube in image) (b). Two patient simulators with same C (Same internal tube) and different value of R (c).

When a higher pressure is applied to the internal cylinder (Figure 4b), a pressure differential is originated. Assuming a negligible wall thickness for the inner cylinder, the height difference between a rest and pressurized condition also provokes a volume differential at the internal cylinder, which can be combined with the Stevin's law to model a value of compliance, as presented in equation 1.

$$C = \frac{\Delta V}{\Delta P} = \frac{A_i}{\rho g} * \left(\frac{1-A_i}{A_e}\right) \qquad \text{[Eq. 1]}$$

To contain the encumbrance and achieve the desired values, the external tube diameter was fixed to 150 mm, whereas the internal diameter was fixed to three values 90, 100, and 120 mm representing respectively C1, C2, and C3.

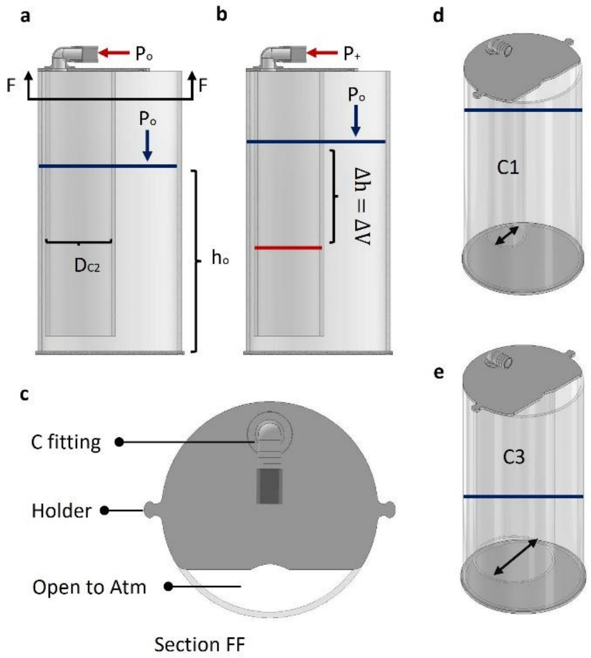

Fig. 4 Rest condition of the compliance (a). Pressurized compliance and accumulation of air (b). Section FF or top view, the holder, internal tube and C fitting are glued together (c). Two compliance models, C1 houses a smaller internal tube than C3, and in addition a different in-rest initial water level is set (d and e)

Airway resistance model

According to the low-pressure assumption taken previously and limiting the value of Reynolds number to less than 2000, the pneumatic resistance element can be based in the Hagen-Poiseuille equation, which has been adapted to calculate the fluid resistance provoked by N parallel channels. Equation 2 shows the final equation and the transformation to pneumatic resistance. By adjusting the bundle length, number of tubes and channels internal diameter it is possible to achieve different values of resistance. Figure 5 summarizes each model value.

$$R_{bundle} = \frac{R_{tube}}{N} = \frac{\Delta P/Q}{N} = \frac{128\mu L}{\pi N D^4} \qquad \text{[Eq. 2]}$$

Where R is the pneumatic resistance, Delta P is the pressure drop at extremities, Q the gas flow rate, μ dynamic viscosity, L the tube length and D the single tube diameter.

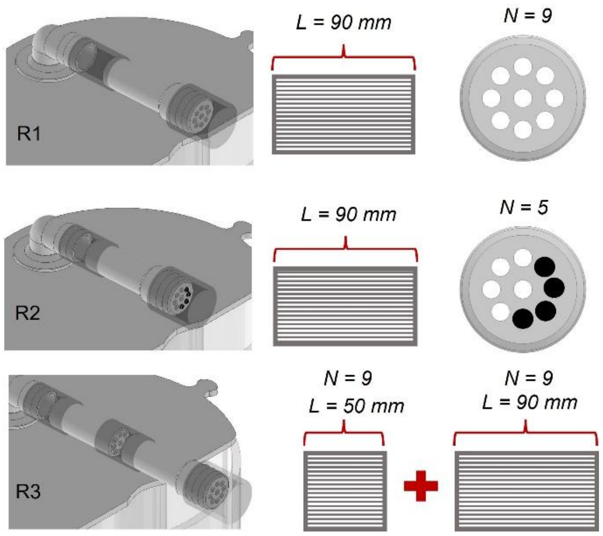

Fig. 5 Three types of airways resistance. R1 represents a patient with mild air flow restriction, R2 medium severity and R3 highly restrictive airway. Resistance elements are connected in series using elastic silicone tubes and in the R2 case some of their internal channels were obstructed.
